# Two Successful Homœopathic Surgical Operations

**Published:** 1893-01

**Authors:** J. H. Allen

**Affiliations:** Logansport, Ind.


### TWO SUCCESSFUL HOMŒOPATHIC SURGICAL OPERATIONS.

J. H. ALLEN, M. D., LOGANSPORT, IND.

CASE A.—Mrs. C., age fifty-one, large and fleshy, of a bilious temperament, subject to rheumatism, and catarrhal colds of the head and lungs; has family history of psora. Has always been treated homœopathically. Six years ago I removed a large tumor from the left scapular region, from which she suffered much pain in using the arm. After the removal of the growth, which was a large one, the wound healed kindly and I discharged her in a few weeks apparently cured. I did not see nor hear from her until one year ago, when she presented herself for another operation, a tumor of a similar nature, which had for the past two years been growing on the left shoulder about three inches from the seat of the former growth, but more anterior, extending toward the neck. I made a diagnosis of the growth similar to the one I had operated upon. When I inquired why she had not applied for treatment sooner, she replied that she knew that nothing but an operation would be of any benefit to her, and as long as she was suffering no inconvenience from it she thought it best not to interfere. It was now causing her considerable pain and annoyance and wished an operation. I should think it would weigh about five pounds. Within the last two months it has grown rapidly and, in appearance, it looked as if it might become a large growth. I assured her that we would not have to operate this time and prescribed Psorinum$^{10m}$, Fincke, followed by no medicine for two months. I do not remember now the symptoms I prescribed it on, but at the end of this time there had developed the following symptoms: feet, hands, and face bloated in the morning. No pain in the tumor now, but can see no change in its size or shape.

She perspires freely under the arms, which has a very offensive odor and which rots her clothing. The feet also perspire a great deal, the odor from which is horribly offensive. Has much rawness between the toes, and, for weeks at a time, the skin peels off them.

Silicea$^{cm}$ was given, and during the period of ninety days, when all the above symptoms disappeared together with the growth. The rapidity with which this growth disappeared under the profound action of this highly potentized Silicea was truly remarkable; it was as much of a surprise to myself as the patient.

What a pleasure it is to cure one of these cases without the aid of the knife. We have in this case a double cure; not only have we cured the abnormal growth, but we have removed that underlying principle that predisposed her from ever in the future being a tumor-producing patient.

Case B.—Mrs. B——, age twenty-seven, brunette, married eight years, has one child, age seven, family history good, father and mother living and well. She has had almost perfect health up to three years ago, when she began to suffer with severe pains in the uterus during the menses and almost constant neuralgia of the ovaries during inter-menstrual period. For the past year she suffered more or less with chills which were very severe, coming on early in the morning and lasting for an hour or more, followed by fever which lasted until about two o'clock in the morning, when she would break into a profuse sweat. She seldom slept until the sweating began. She has emaciated rapidly within the past six months of her illness. Her weight has decreased in the past two months thirty pounds. During the past two months she has been confined to her bed and treated for lung trouble, though she has neither cough, soreness, or any pain in the lungs, nor is there any history of a lung trouble in the family. But on a careful examination of her case I find a cancer of the uterus in an advanced stage and that of a sycotic nature. My opinion was confirmed by Dr. E. R. Sawyer, of Kokomo, Ind. The whole right anterior half of the body of uterus was involved, it also extended, in the form of a band, around the rectum, which had begun to ulcerate and is discharging at each stool bloody pus and mucus. The bladder is being infringed upon somewhat, which causes urination to be more frequent and painful. The chills come every day, followed by fever and profuse and debiltating sweats, for which China was prescribed. The menses have not made their appearance for three months. Previous to the suppression of the menses she had been treated for a profuse acrid leucorrhœa by medicated injections. The urethral orifice is very much swollen, vagina sensitive, ovaries somewhat enlarged and sensitive to pressure. There is a slight watery discharge from the uterus, which produces intense pruritus, and which is relieved by frequent bathing, and has an odor of decayed fish. Medorrinum was prescribed in the CM potency, followed by no medicine for two months. Marked improvement followed this prescription ; in one week all her pains had ceased. The ovaries are less sensitive and the symptoms much better in every way. Menstruation came on at the end of the second week after this prescription—though painful, it seems quite normal otherwise. The pruritus has ceased and she cannot detect that peculiar fishy odor nearly as plainly. Chills do not come on nearly as often ; for the past four days has had none, which is the first time they have remained away so long for six months. At the end of the eighth week I gave another powder, as she was not as well. From that time on she rapidly grew better. The face grows less pinched and the color better. Her eyes have a less hollow look, and the dark circles are leaving them. The sweats are much less, and she sleeps all night, feeling less exhausted in the morning. No more medicine was given for sixty days, when a severe diarrhœa set in calling for Podophyllum, which was given in water, followed by no medicine for one week, when Conium was prescribed, based upon the symptoms of the mammary gland during the menstrual period.

For the past two months she has done all her own house work, and is improving every day. On examination I find the growth has disappeared and the hypertrophied walls of the uterus have almost resumed their normal condition. A slight thickening remains in the uterine walls, but the tenderness and soreness have all disappeared. I prescribed for her before I left for this meeting what I considered in her case a good prescription, NO MEDICINE.

### ADDITIONAL REPORT OF BOARD OF CENSORS.

James Ferrier, M. D., of New York, Alfred J. Norman, M. D., of Rochester, and Marvin A. Custis, M. D., of Washington, D. C., were elected to junior membership, the Secretary casting the ballot for each.

---

### REPORT OF COMMITTEE ON VACCINATION.

In considering the resolutions on Vaccination, your Committee deems the first one of so great importance to every member of this Association that we would suggest that it be referred to a special committee of three, whose duty it shall be to make a general report on the subject of vaccination at the next annual meeting.

Regarding the second resolution, your Committee unanimously recommends its adoption.

H. Hitchcock, *Chairman.*
A. R. Morgan,
W. P. Defriez,

The second resolution, as follows, was adopted :

*Resolved,* That compulsory vaccination is unjustifiable and contrary to the rights and privileges of the people.

